# Electrode Surface Area Impacts Measurement of High Frequency Oscillations in Human Intracranial EEG

**DOI:** 10.1109/TBME.2024.3416440

**Published:** 2024-10-25

**Authors:** Kavyakantha Remakanthakurup Sindhu, Marco A. Pinto-Orellana, Hernando C. Ombao, Aliza Riba, Donald Phillips, Joffre Olaya, Daniel W. Shrey, Beth A. Lopour

**Affiliations:** Department of Biomedical Engineering, University of California, USA.; Department of Biomedical Engineering, University of California, USA.; Statistics Program, King Abdullah University of Science and Technology, Saudi Arabia.; Division of Neurology, Children’s Hospital of Orange County, USA.; Division of Neurology, Children’s Hospital of Orange County, USA.; Division of Neurosurgery, Children’s Hospital of Orange County, USA; Department of Neurosurgery, University of California, USA.; Division of Neurology, Children’s Hospital of Orange County, USA; Department of Pediatrics, University of California, USA.; Department of Biomedical Engineering, University of California, Irvine, CA 92697 USA

**Keywords:** Biomarker, epilepsy, epilepsy surgery, fast ripples, ripples

## Abstract

**Objective:**

High-frequency oscillations (HFOs) are a promising prognostic biomarker of surgical outcome in patients with epilepsy. Their rates of occurrence and morphology have been studied extensively using recordings from electrodes of various geometries. While electrode size is a potential confounding factor in HFO studies, it has largely been disregarded due to a lack of consistent evidence. Therefore, we designed an experiment to directly test the impact of electrode size on HFO measurement.

**Methods:**

We first simulated HFO measurement using a lumped model of the electrode-tissue interaction. Then eight human subjects were each implanted with a high-density 8×8 grid of subdural electrodes. After implantation, the electrode sizes were altered using a technique recently developed by our group, enabling intracranial EEG recordings for three different electrode surface areas from a static brain location. HFOs were automatically detected in the data and their characteristics were calculated.

**Results:**

The human subject measurements were consistent with the model. Specifically, HFO rate measured per area of tissue decreased significantly as electrode surface area increased. The smallest electrodes recorded more fast ripples than ripples. Amplitude of detected HFOs also decreased as electrode surface area increased, while duration and peak frequency were unaffected.

**Conclusion:**

These results suggest that HFO rates measured using electrodes of different surface areas cannot be compared directly.

**Significance:**

This has significant implications for HFOs as a tool for surgical planning, particularly for individual patients implanted with electrodes of multiple sizes and comparisons of HFO rate made across patients and studies.

## I. Introduction

High-frequency oscillations (HFOs) are transient bursts of electrophysiological activity with peak frequencies greater than 80 Hz, associated with both normal physiological processes and epilepsy [1]. They have been studied extensively in the past two decades as prognostic biomarkers of surgical outcome in patients with epilepsy [2]. The first HFOs detected in human intracranial EEG (iEEG) were recorded at very small spatial scales, using microwires with diameters in the 40-micron range [3], [4]. HFOs were subsequently detected in recordings from much larger clinical depth electrodes, approximately 1 mm^2^ in area [5]. Today, they are recorded using electrodes with contact areas that range from 0.003 mm^2^ to 20 mm^2^, with a myriad of different geometries [6], [7]. There is also increasing evidence that this high-frequency electrophysiological activity is measurable using non-invasive scalp EEG electrodes [8]. However, all such events are singularly deemed to be “HFOs,” regardless of the spatial scale at which they are measured. It is unknown if the events detected at different spatial scales represent the same neural phenomenon or have equivalent clinical relevance [9].

This variability in the size of recording electrodes used for HFO detection is a potential confounding factor in current studies. It has been shown that HFOs occur more frequently in the seizure onset zone (SOZ) [10], [11] and are a promising tool for surgical planning, as the removal of HFO-generating brain regions is associated with positive long-term outcomes [9], [12], [13], [14]. However, despite strong associations at the group level, HFOs have not been shown to be reliable predictors of outcome for individual patients [12], [13]. In part, this discrepancy could be due to equating results from a wide range of electrode contact surface areas with one another, often within the same patient or research study. If electrode size impacts the measurement of HFOs, e.g., if large electrodes over- or underestimate the HFO rate, it would significantly impact study results.

Some in silico studies have shown differences in EEG measurements that are a function of electrode size, like voltage sensitivity [15] and correlation between electrodes [16]; others showed no significant differences [17]. Studies that analyzed the effect of electrode size on the signal-to-noise ratio (SNR) of extracellular recordings also presented conflicting results [18], [19], [20], [21]. A previous in vivo study done by our group showed that EEG amplitude and power decreased when the size of recording electrodes increased, and interictal spikes had higher SNR in the smallest electrodes [22]. Electrode size also affects the contact impedance of EEG electrodes [23], [24] which can impact recordings.

There has been no consensus on how these differences in EEG measurements translate to HFO measurements. A small number of studies have examined the effect of electrode size on HFO measurement, and they have offered conflicting results. Worrell et al. reported that microwires (40 *μ*m diameter) recorded more fast ripples (250–500 Hz) than macro-electrodes (2.3 mm diameter)[25]. Another study found a similar trend, with high density electrocorticography (HD-ECoG) electrodes of 2.3 mm diameter recording more fast ripples than larger ECoG electrodes of 5 mm diameter [26]. However, Chatillon et al. reported no such difference in the detectability of fast ripples between electrodes with diameters of 0.2 mm and 0.8mm [7]. For ripples (80–250 Hz), small differences in rate (number per minute) were reported for sizes ranging from 0.02 to 0.09 mm^2^, but were deemed to be not clinically significant [7], [27]. In an intra-operative ECoG study using 2.3 mm and 5 mm diameter electrodes, it was suggested that fast ripple rates delineated the SOZ more accurately when electrodes with smaller contact areas and shorter inter-electrode distances were used [26]. Two studies reported differences in the average peak frequency of ripples for different electrode sizes. Worrell et al. 2008 reported that the average peak frequency for microelectrodes was higher than for standard clinical depth electrodes, 143.3 Hz compared to 116.3 Hz [25]. Blanco et al. 2011 found no such frequency difference for subdural grids, but reported the same difference for depth electrodes when compared to microelectrodes [10]. Modur et al. 2011 reported no difference in characteristics of HFOs recorded using subdural electrodes (1.1 mm in diameter) compared to depth electrodes (2.3 mm in diameter) [28].

However, in these studies, the electrodes of different sizes were adjacent to one another, in spatially distinct brain regions, or in different subjects altogether. Because characteristics of HFOs vary significantly between subjects and brain regions [9], [29], any variations found by these studies cannot be solely attributed to the differences in electrode size. Further, no current methods enable separation of the effects of electrode size, regional variation, and differences in epileptiform activity. This impedes a direct comparison of HFO characteristics between different electrode sizes.

To address this, we measured HFOs using three different sizes of subdural iEEG electrodes implanted in the human brain. Unlike previous studies, we recorded from the exact same region of brain tissue using all three electrode sizes; this was accomplished using a technique developed by our group [22]. We then directly compared the HFO characteristics of rate, amplitude, duration, and peak frequency when measured with electrodes of different effective surface areas. We also calculated the rate of HFOs per unit area of electrode to account for the fact that larger electrodes measure from a larger portion of cortical tissue. To the best of our knowledge, this is the first study to record iEEG from the same tissue location for all electrode sizes, which is paramount to calculating this metric.

## II. Methods

### A. Model of HFO Measurement

**Fig. 1.**
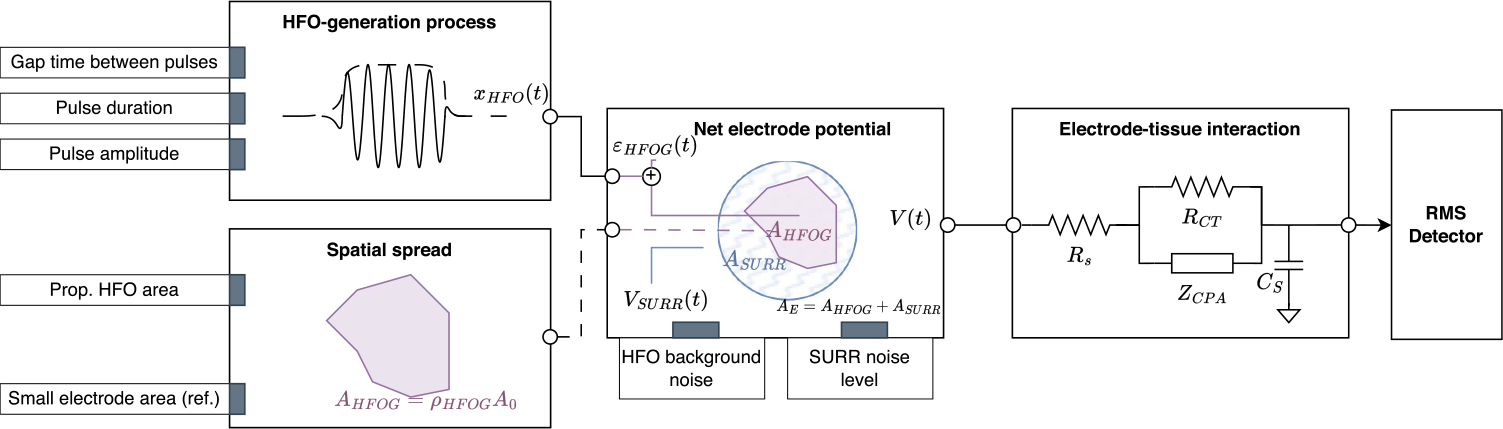
Model workflow with the different simulation components. The input parameters are denoted as grey blocks in their respective processes.

To develop a model for the impact of electrode size on HFO measurement, we combined simulated iEEG data with a lumped model of the electrode-tissue interaction (Fig. 1). We briefly describe the model here. Full details are reported in Appendix A. The simulated iEEG contained trains of HFOs that were defined by distributions of event amplitude, duration and inter-event intervals. The net electrical potential measured by the electrode was a function of the electrode surface area and the proportion of that area covered by HFO-generating tissue. It was assumed that each HFO would occur within a noisy background signal and that noise would also be contributed by the surrounding non-HFO-generating tissue. To estimate the signal resulting from measurement of the simulated iEEG with a platinum electrode, we adapted the lumped model from [30] that was used to simulate HFOs in a mouse hippocampus using a kainate model of temporal lobe epilepsy. To the best of our knowledge, this is the first time the model has been applied to human intracranial EEG signals.

Using this model, we simulated 5000 iEEG signals, each 20 minutes long at a sampling frequency of 1000Hz. Three different electrode surface areas were simulated, chosen to match the human iEEG recordings. HFOs were detected using a previously validated automated algorithm based on the root-mean-square (RMS) amplitude [31]. In short, the RMS amplitude of the iEEG was calculated in 3 ms sliding windows, and segments in which the RMS amplitude exceeded a threshold for a minimum duration of 6 ms were marked as events of interest. Events with at least six peaks crossing a second threshold were defined to be HFOs. Both detection thresholds in the algorithm were set to three standard deviations above the mean of the rectified, filtered signal. Lastly, the HFO rate per minute *r*_min_ was calculated for each sequence, and the duration and amplitude were calculated for each detected HFO.

### B. Patients and Data

This study was approved by the Institutional Review Board of the Children’s Hospital of Orange County (Protocol #1812117, approved January 14, 2019). We recruited eight pediatric subjects with medically intractable epilepsy and focal seizures that were undergoing pre-surgical invasive monitoring (Supplementary Table I). Each subject had a high-density (HD) 8×8 subdural grid of iEEG electrodes (Ad-Tech FG64C-MP03X-000) implanted, in addition to any other standard intracranial electrodes deemed necessary by the clinical team. However, in this study, we analyzed only the data from the HD grid. In five subjects, the HD grid recorded iEEG from the brain region that was clinically determined to be the SOZ. Electrodes in the HD grid had a surface area of 1.08 mm^2^ and center-to-center pitch of 3 mm. This is approximately one fourth of the size of standard subdural grids, which typically have 4 mm^2^ electrode surface area and 10 mm pitch.

**Fig. 2.**
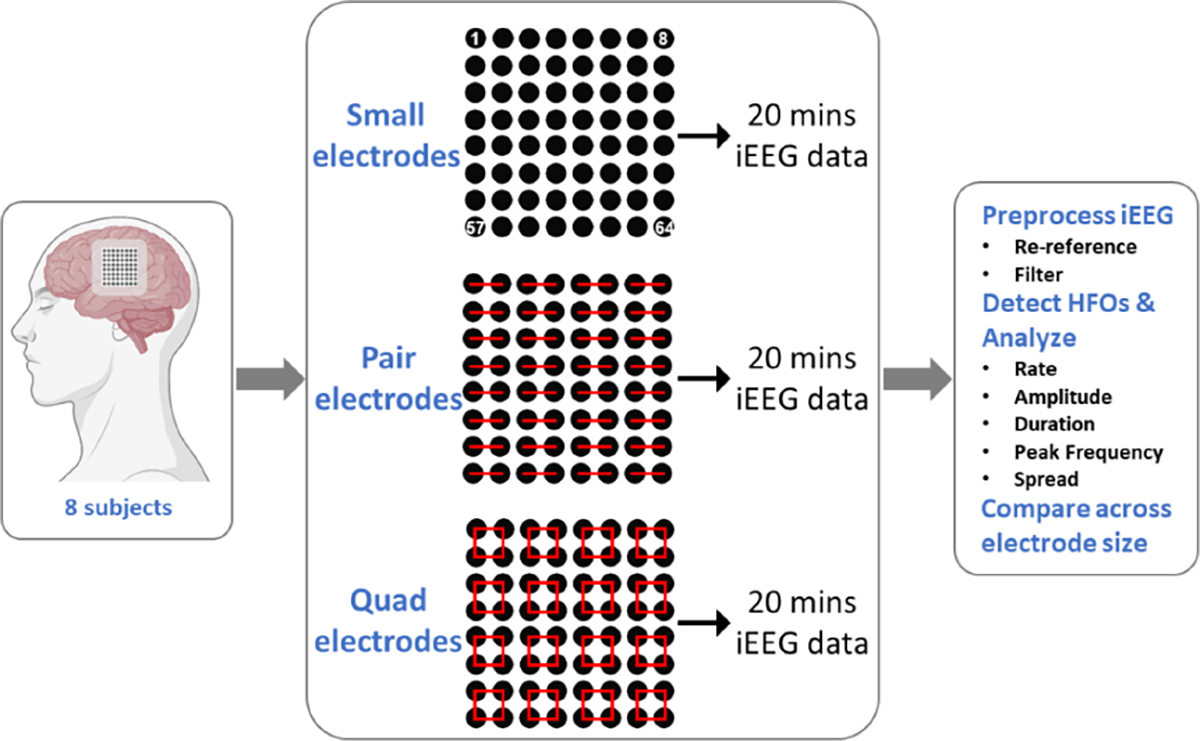
Increasing surface area via shorting of electrodes.

After implantation, we modified the recording setup for the HD grid electrodes to alter the electrode surface area as described in [22]. Briefly, this was done by electrically shorting adjacent electrodes in groups of two or four using jumper wires at the junction box, outside the head (Fig. 2). Shorting adjacent electrodes effectively averages the electrical activity of their underlying brain tissue [16], [32], as would be recorded by a larger electrode in the same location. Thus, we could mimic larger surface areas of 2.16 mm^2^ and 4.32 mm^2^ when shorting together two and four adjacent electrodes, respectively. We recorded approximately 20 minutes of iEEG for each of the three different electrode surface areas (which we will refer to as “small”, “pair,” and “quad” electrodes) while the subjects were sleeping. Whenever possible, the experiment was conducted in the evening one to two days after implantation (Supplementary Table I), and at least two hours after the most recent seizure had occurred. Note that the effective surface area of a “quad” electrode is roughly comparable to that of a standard clinical macro-electrode.

### C. Preprocessing and HFO Detection

The recorded iEEG data were re-referenced to the common average of the HD grid. These data were then bandpass filtered in the ripple (R, 80–250 Hz) and fast ripple (FR, 250–500 Hz) frequency bands and notch filtered at the odd harmonics of 60 Hz to remove line noise. Then, the data were divided into one-minute segments for automatic HFO detection using the same algorithm described in Section II.A [31]. False positive detections were rejected using the methods outlined in Gliske et al. 2016 for removing artifacts present in the common average reference and those caused by fast transients or DC shifts [33].

### D. Calculation of HFO Properties

The average rate of HFOs (number per minute) was calculated over the duration of the recorded iEEG for each channel in the ripple and fast ripple bands separately. In each subject, we thus obtained 64, 32, and 16 values of HFO rates for the small, pair, and quad electrode configurations, respectively. These HFO rates were then compared within each subject using a Wilcoxon rank sum test [34], separately for each frequency band (R and FR). Then, because each electrode size measured from a different amount of tissue, we did a second comparison where we normalized HFO rate for electrode size by calculating the global rate per unit area of tissue. The global rate was defined as the total number of unique events detected by the entire HD grid (counting any overlapping events only once), divided by the total duration of data in minutes. The global rate per area was defined as the ratio of the global rate to the summed area of all 64 electrodes. This metric was used to compare the three electrode sizes across all subjects using the Wilcoxon rank sum test.

We also assessed the impact of electrode size on three other HFO characteristics: amplitude, duration, and peak frequency. The amplitude of each HFO was estimated as the average value of the upper Hilbert envelope of the signal over the duration of the event [35]. The peak frequency of an HFO was defined as the frequency for which the magnitude of the Fourier transform of the signal was maximal, following a whitening process to attenuate the power of the low-frequency components [36]. The Wilcoxon rank sum test was used to test the dependence of HFO amplitude, duration, and peak frequency on electrode size.

Finally, we measured the spatial extent of each HFO using the “spread” (S), defined as the number of electrodes in which an HFO was simultaneously observed. HFOs that were only detected in one electrode and did not overlap in time with a detected HFO in any other electrode had S=1, while events that were observed in more than one electrode had S≥2. We calculated the spread for single, pair, and quad electrodes and compared their distributions in the ripple and fast ripple bands.

## III. Results

### A. Simulations of HFO Measurement Suggest That Rate and Amplitude Will be Affected by Electrode Size

**Fig. 3.**
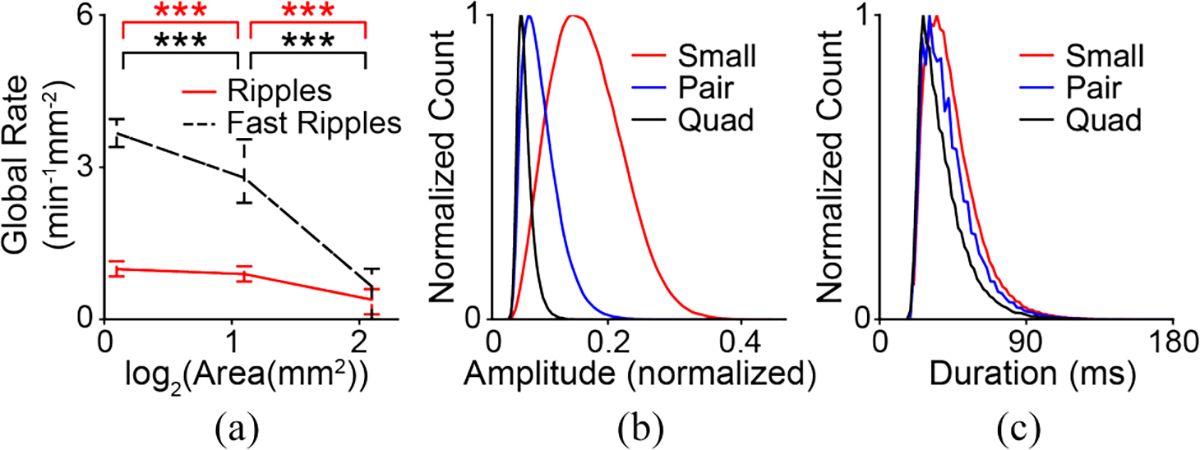
Simulation results for the HFO measurement model. (a) HFO rate per electrode surface area, (b) HFO amplitude, and (c) HFO duration for fast ripples measured by electrodes of three different surface areas.

Based on 5000 simulated iEEG signals containing HFOs, the model suggested that the rate of detected fast ripples will decrease significantly as electrode size increases (Fig. 3(a)). Moreover, fast ripple amplitude will decrease as electrode size increases (Fig. 3(b)), but duration will be unaffected by electrode surface area (Fig. 3(c)). The model suggested that ripples would exhibit similar decreases in rate (Fig. 3(a)) and amplitude (Supplementary Fig. S1).

### B. HFO Rate Decreased With an Increase in Electrode Size

**Fig. 4.**
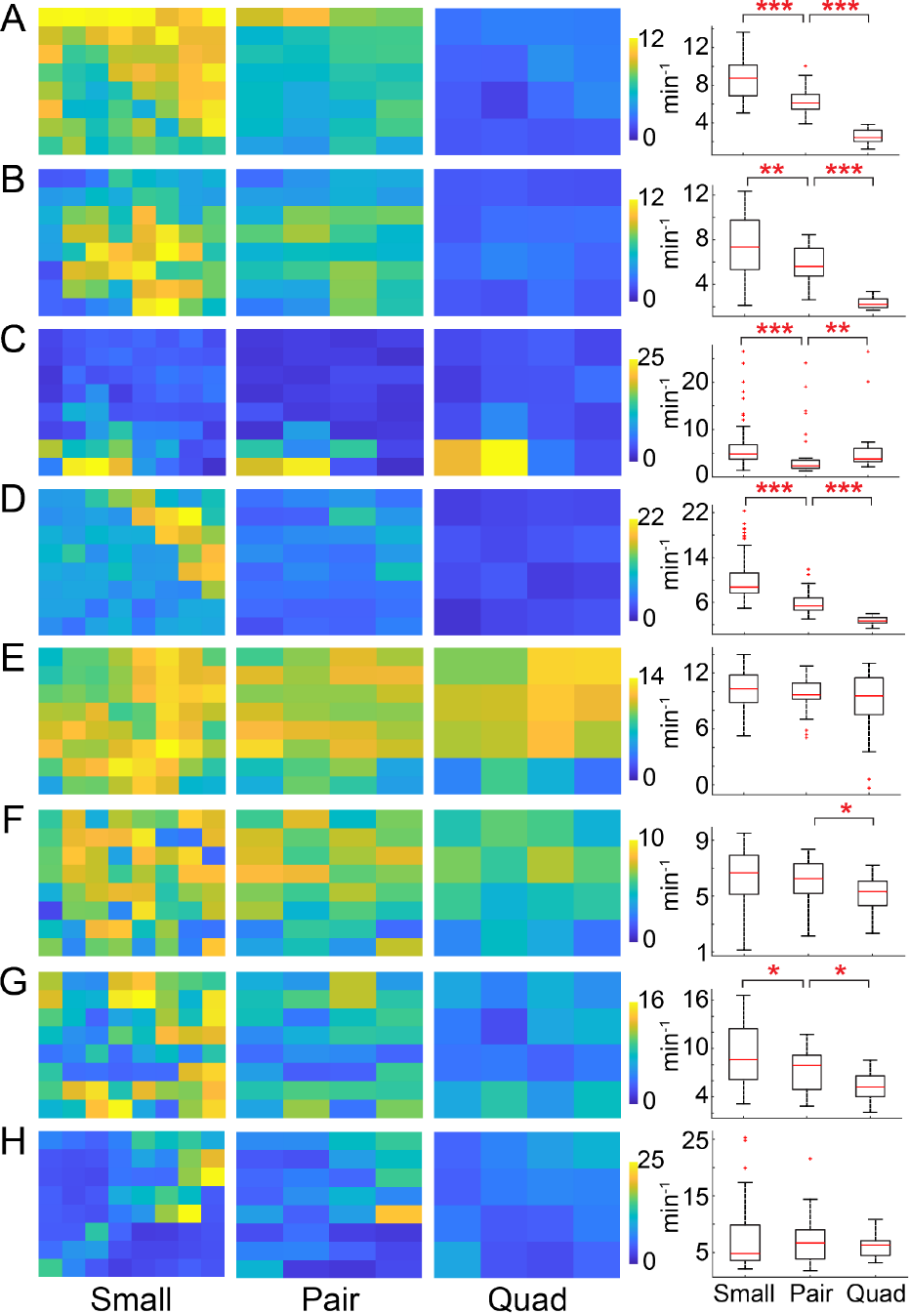
Fast ripple rates across the HD subdural grid for Subject 1 (subfigure A) through Subject 8 (subfigure H). Heatmaps of fast ripple rates for small, pair, and quad electrodes are shown on the left of each subfigure. The right side of each subfigure shows boxplots comparing fast ripple rates across all electrodes for the three electrode sizes. * indicates p<0.05, ** indicates p<0.01 and *** indicates p<0.001.

Fig. 4 shows heatmaps of fast ripple rates for all eight subjects across all three electrode sizes. In general, the warmest colors, denoting the highest rates, are seen in the small electrodes. For some subjects, regions of high rate can be easily localized using any electrode size (e.g., Fig. 4(c) and (e)). In others, they can only be delineated using the smaller electrodes (e.g., Fig. 4(d) and (h)). The average FR rate in the small electrodes was significantly higher than in pair electrodes in five subjects (p<0.05) and higher in the pair electrodes compared to quad electrodes in six subjects (p<0.05, Fig. 4). In the ripple band, the average HFO rate was significantly higher in small electrodes compared to pair electrodes in five subjects and in pair electrodes compared to quad electrodes in three subjects (p<0.05 for both comparisons).

**Fig. 5.**
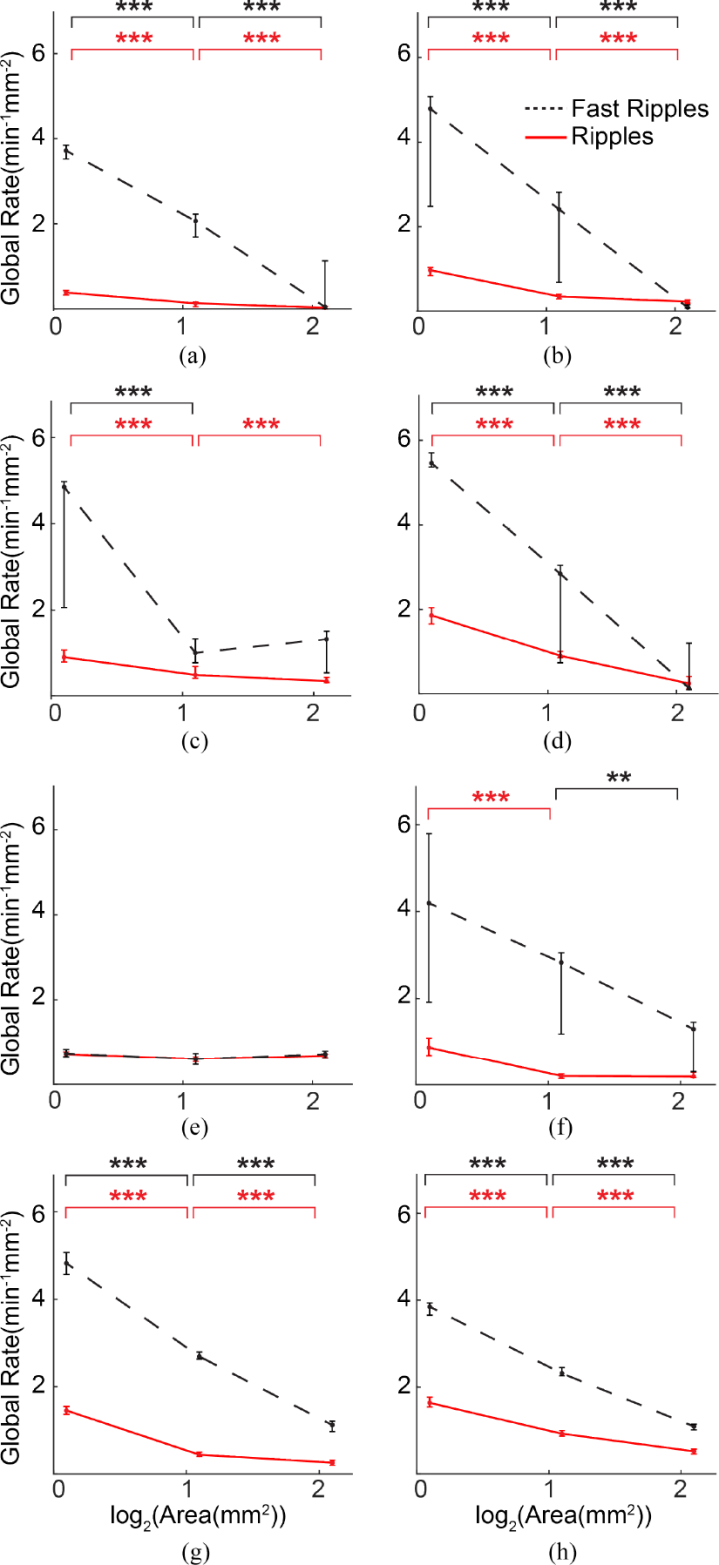
Global HFO rate per area of electrode for Subject 1 (subfigure A) through Subject 8 (subfigure H). The dots and error bars represent the median and 25th and 75th percentile values, respectively, of global HFO rate per area calculated in one-minute epochs. Each subfigure shows data obtained from small, pair, and quad electrodes, summed across the entire HD grid and normalized by total area. Results for ripples (solid red line) and fast ripples (dashed black line) are shown. * indicates p<0.05, ** indicates p<0.01 and *** indicates p<0.001.

However, it could be considered inaccurate to directly compare the HFO rates from electrodes of different sizes, as shown in the right-hand column of Fig. 4, because larger electrodes measure from greater volumes of tissue. To address this, we measured the total HFO rate over the area of the grid for each electrode size, while ensuring that events detected simultaneously in multiple electrodes were only counted once (Section II-D). After making these corrections, the global rate per area of HFOs decreased with increasing electrode size in both frequency bands (Fig. 5). Note that this is consistent with predictions made by the HFO measurement model in Fig. 3(a).

### C. Small Electrodes Recorded More Fast Ripples Than Ripples

**Fig. 6.**
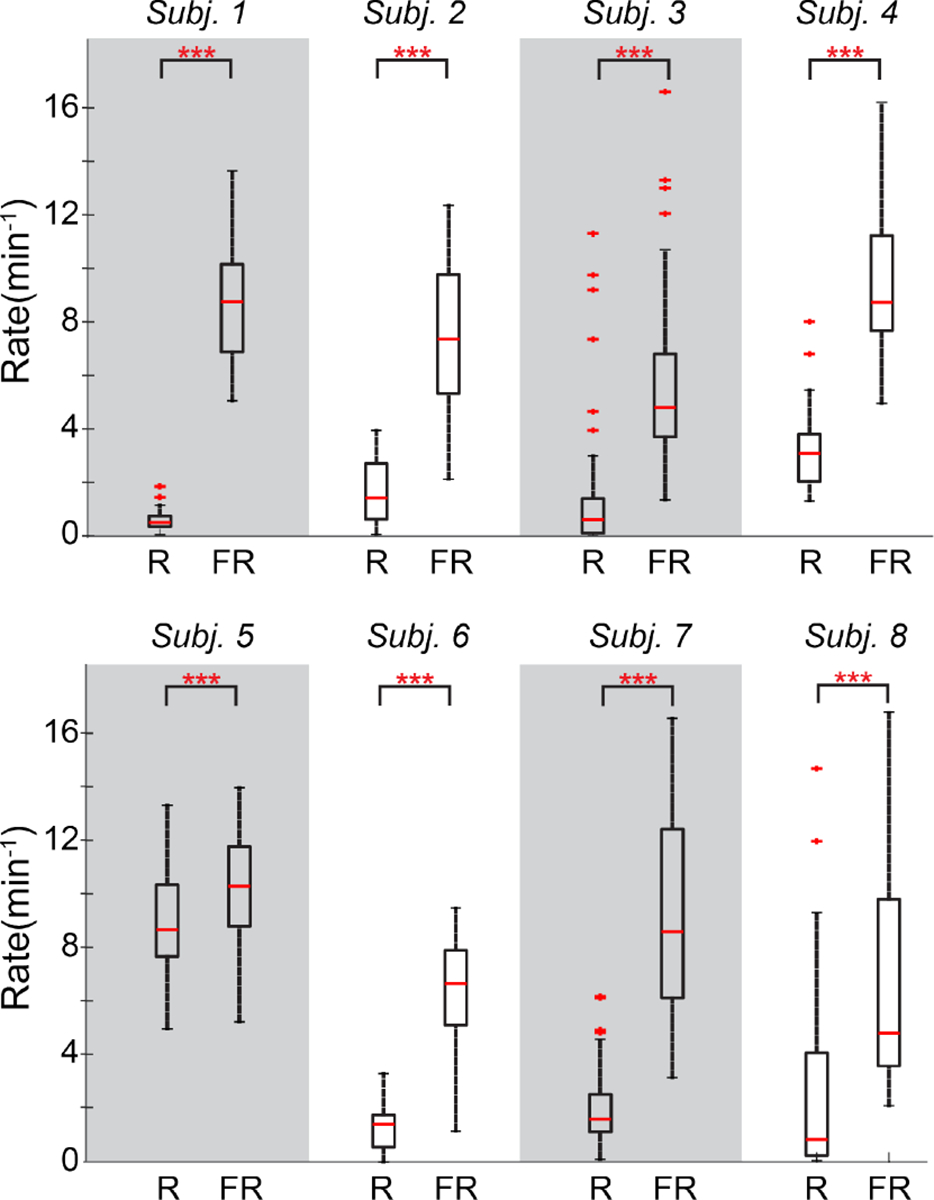
Boxplots showing the channel-wise HFO rates for small electrodes within each subject for ripples (R) and fast ripples (FR). * indicates p<0.05, ** indicates p<0.01 and *** indicates p<0.001.

The small electrodes recorded more fast ripples than ripples for all subjects (p<0.001) (Fig. 6), which is contrary to results that have been reported for standard clinical electrodes [14], [37], [38], [39]. This trend was also observed in most subjects in the pair and quad electrodes (Supplementary Fig. S2), but the difference between R and FR rates was smaller for the larger electrodes.

### D. HFO Amplitude Decreased as Electrode Size Increased

**Fig. 7.**
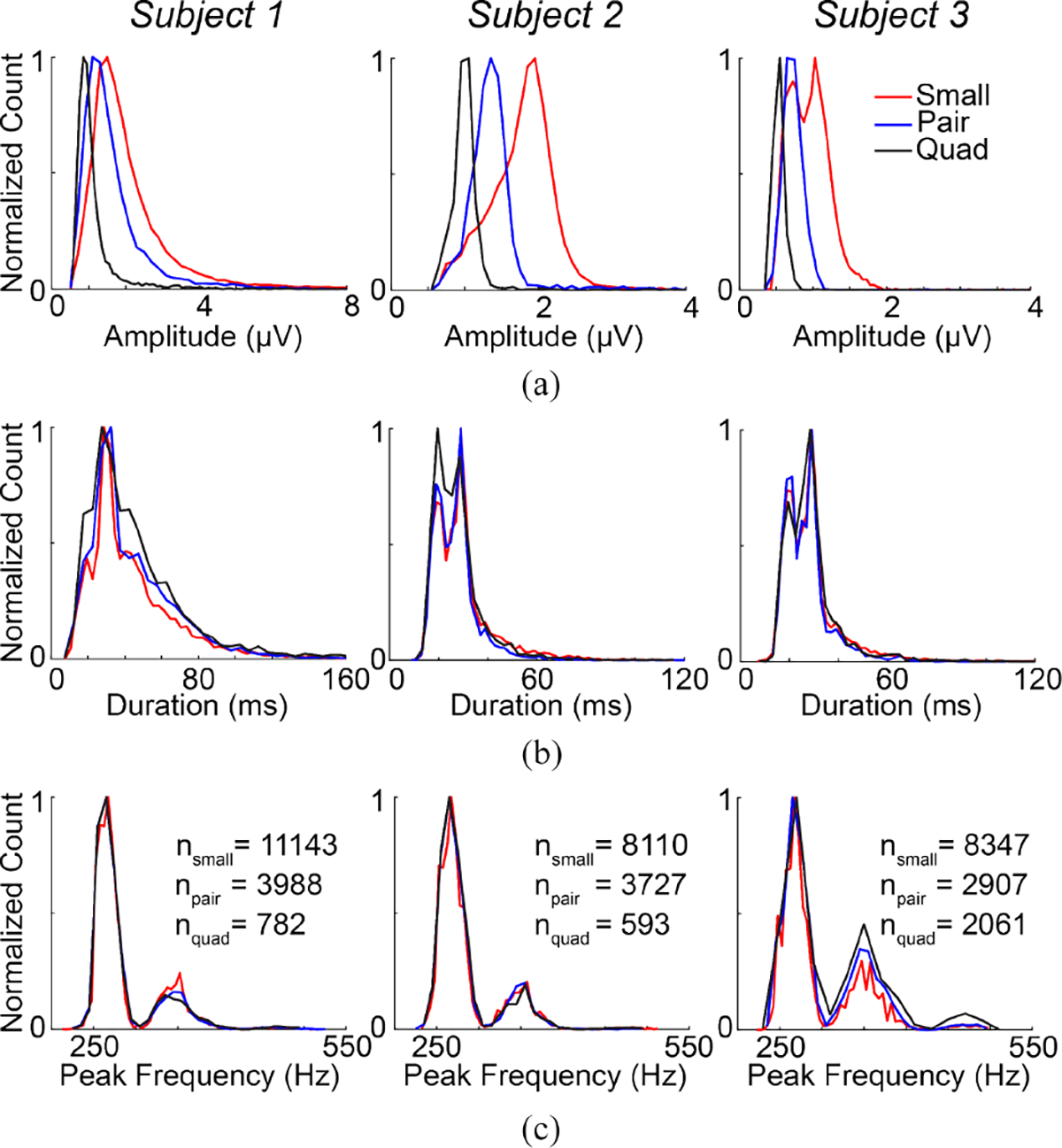
Histograms of (a) amplitude, (b) duration, and (c) peak frequency for fast ripples in small, pair, and quad electrodes in three representative subjects. The normalized counts in the histograms were obtained by dividing each count value by the highest value for each property.

The amplitude of ripples consistently decreased with increasing electrode size, with small electrodes exhibiting significantly larger amplitudes than pair electrodes in all subjects (p<0.001) and pair electrodes exhibiting significantly larger amplitudes than quad electrodes in seven of the eight subjects (p<0.001). A similar relationship was observed in fast ripples, with small electrodes exhibiting significantly larger amplitudes than pair electrodes in seven subjects (p<0.001) and pair electrodes exhibiting significantly larger amplitudes than quad electrodes in all eight subjects (p<0.001) (Fig. 7(a) and Supplementary Fig. S3). HFO duration and peak frequency did not show any such consistent trends across electrode sizes (Fig. 7(b) and (c) and Supplementary Fig. S4 and S5). Again, these results are consistent with the predictions made by the HFO measurement model in Fig. 3(b) and (c).

### E. Most HFOs Exhibited Single-Electrode Spread for All Electrode Sizes

**Fig. 8.**
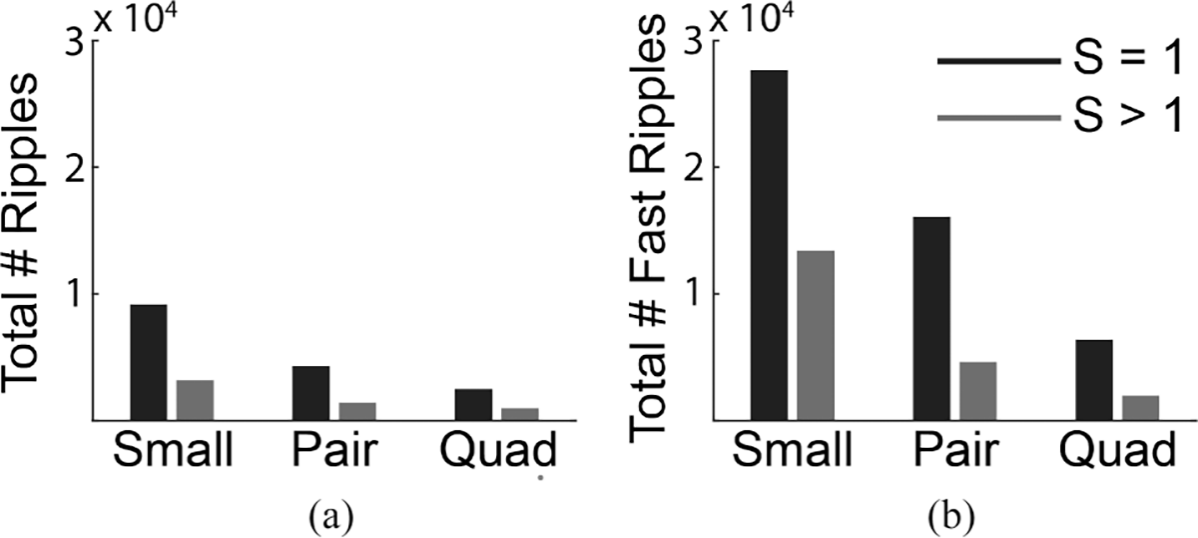
Distribution of events with spread S = 1 and S > 1 across electrode sizes for (a) ripples and (b) fast ripples, summed across all subjects.

Across all subjects, 71% of recorded HFOs had S=1, implying that HFOs that are localized to smaller regions of tissue occur more frequently than larger HFOs (Fig. 8). We would expect that all events with S>1 at one electrode size (e.g., small electrodes) would be measurable using the next largest electrode size (e.g., pair electrodes). In addition, some high-amplitude HFOs with small spatial extent (S=1) will survive the spatial averaging and still be measurable using the next largest electrode size. Consistent with this idea, we found that the pair electrodes recorded approximately twice as many HFOs as the number of HFOs with S>1 detected by small electrodes. A similar relationship was found between pair and quad electrodes, for both ripples and fast ripples.

## IV. Discussion

This is the first study to record HFOs in iEEG using electrodes of different sizes, with all electrodes placed over the same region of neural tissue. This enabled us to isolate electrode size as a variable, as we eliminated the confounding factors associated with placing the electrodes of different sizes in different brain regions or different subjects.

We found that the rate and amplitude of HFOs significantly decreased as electrode size increased. It is likely that two interrelated factors contributed to this result. First, as electrode size is increased, greater volumes of neural tissue are sampled by each electrode. In theory, this could cause an increase in the rate of detected HFOs for larger electrodes. For this reason, it was critical to account for electrode surface area when comparing the HFO rates. Here, we did that by calculating the global HFO rate across the fixed area of the HD grid, and we found that this measurement of rate reliably decreased as electrode size increased (Fig. 5). To confirm that the higher rates for smaller electrodes were not due to cases in which a single HFO co-occurred in multiple electrodes, those co-occurring events were identified and counted only once. Note that, when these corrections were not made, the relationship between HFO rate and electrode size was less clear (Fig. 4.), which could explain results from prior studies.

The second factor is that, as electrode size increases, the single EEG measurement for that electrode is the result of averaging over a larger region of tissue. HFOs are believed to have relatively small neural generators compared to the size of standard iEEG electrodes [40]. Therefore, as electrode size increases, HFO generators comprise a lesser proportion of the sampled tissue, which decreases the signal-to-noise ratio for the HFO compared to the background neural activity. In many cases, this leads to a lack of detection, thereby lowering the measured HFO rate. Our results were consistent with this theory in all subjects, except for Subject 5.

Subject 5 exhibited high HFO rates across all electrodes, showing no significant differences in rate between the different electrode sizes (Figs. 4 and 5). Unlike the other subjects, most of the HFOs recorded from Subject 5 were spread over many electrodes and were thus detected at all spatial scales. This is evidenced by the low global HFO rate in Fig. 5 (~1/min/mm^2^ after removal of co-occurring events), compared to the raw HFO rate of ~10/min in Fig. 4. In all other subjects, the proportion of duplicate events that occurred due to high spatial spread depended on electrode size (Fig. 8).

Studies using standard clinical depth and subdural electrodes have reported higher rates of ripples than fast ripples [14], [37], [38], [39]. In the small electrode configuration, our results contrast with this, as significantly higher rates of fast ripples than ripples were detected (Fig. 6). However, as the size of the electrode increased, the fast ripple rate fell more quickly than the ripple rate (Fig. 5), thus approaching the relationship reported in prior literature for large electrode sizes. This result suggests that measurement of fast ripples may be facilitated by the use of smaller electrodes.

Accurate localization of the so called “epileptogenic zone,” the minimum tissue to be resected to achieve post-surgical seizure freedom, has been a subject of epilepsy research for decades. Removal of brain regions exhibiting high rates of HFOs has been shown to be correlated with good post-surgical outcome [14], [37], [41]. We found that, in some subjects, delineating regions of high HFO rate was possible using any of the three electrode sizes (Fig. 4(c) and (e)), while in others, it was only clear when using the smaller electrodes (Fig. 4(d) and (h)). This suggests that electrode size may impact the efficacy of HFOs as prognostic biomarkers of post-surgical outcome in some patients, and further research in this direction is needed to accurately model this effect.

Surgical candidates with refractory epilepsy are often implanted with multiple types of electrodes during their phase two evaluation. Our results suggest that high HFO rates measured with small electrode sizes could be erroneously attributed to the presence of epileptogenic tissue in the corresponding brain region, resulting in ill-founded surgical decision making. Moreover, automated HFO detection algorithms are being increasingly employed, most of which use some form of amplitude thresholding [42]. Because HFO amplitude is also affected by electrode size, threshold optimization for each electrode type may be beneficial.

The methods used in this study have similar limitations to those discussed in the original description of the electrode-shorting method [22]. Briefly, the shorted electrodes are spaced 3 mm apart, and therefore, the ensemble electrode is not contiguous as it would be in the case of a larger electrode placed at the same location. However, we previously compared measurements from the electrically-shorted pair electrodes to measurements from a larger single electrode with an equivalent surface area, and we found no statistically significant differences between the two [22]. We were unable to use a grid with a smaller inter-electrode distance as we were limited to FDA-approved electrodes. Further, the recordings from the three configurations were taken sequentially and not simultaneously. This could introduce differences between the recordings owing to the non-stationarity of iEEG signals. To minimize this effect, the recordings for the three electrode sizes were typically done sequentially within a 60 to 90-minute time window. Additionally, we used approximately 20 minutes of data per recording configuration and detected HFOs in 1-minute windows. The rates of HFOs were observed to be fairly consistent over time (note also the consistency between the heatmaps for small, pair, and quad electrodes in Fig. 4), indicating that effects due to recording sequential epochs were minimal.

**Fig. 9.**
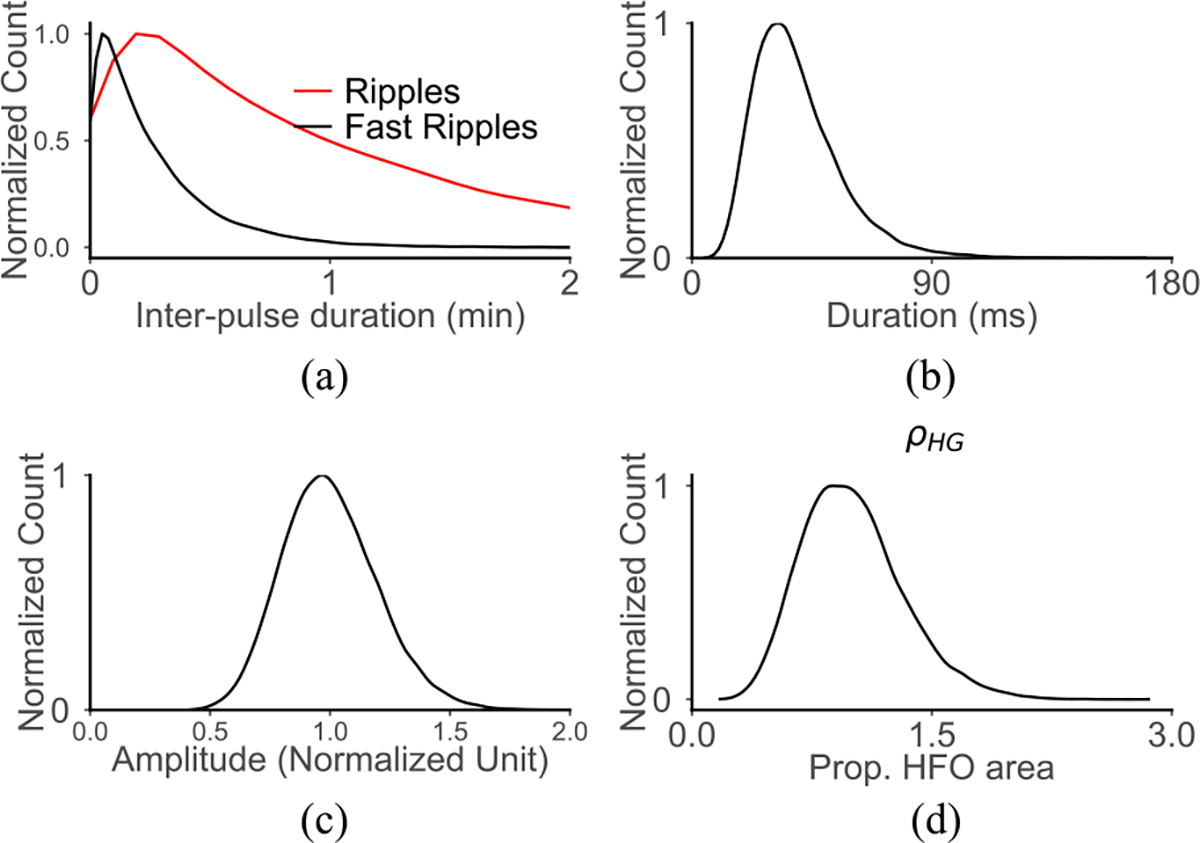
Distribution of the (a) inter-pulse gap time s, (b) pulse duration d, (c) pulse amplitude K, and (d) the HFO proportion $\rho_{HFOG}$.

There were also limitations to the model we developed to simulate HFO measurement. First and foremost, the true nature of the electrophysiological signals underlying HFOs has not yet been characterized. Prior studies have reported measurements of features such as peak frequency [43], amplitude [44], and duration [45]; we used these to inform our model, but with the knowledge that each result was a function of the electrodes used to perform the measurements. Similarly, no study has reported evidence of a characteristic size or spatial extent of HFOs. In fact, studies using electrodes ranging from intracranial microwires [46] to scalp EEG electrodes [47], [48] have reported HFOs that are isolated in a single electrode and not visible in adjacent electrodes. Here, we address this by measuring the HFO-generating area in proportion to the electrode size, with values ranging from almost zero to three times the size of the measuring electrode. This covers the full range of very small events that are highly unlikely to be visible in the iEEG measurement, to very large events with a high likelihood of detection. However, this aspect of the model could be improved if future studies address this gap in the literature. It is also worth noting that some of our results were independent of the initial distribution chosen for the simulated HFOs; compare, for example, the underlying distribution of HFO amplitude in Fig. 9(c) to the amplitude of simulated detected HFOs in Fig. 3(b). Despite initially assuming a symmetric distribution (that resembles a Gaussian shape), the measured amplitude distribution is skewed towards lower values, matching the human HFO measurements in Fig. 7(a).

## V. Conclusion

Overall, these results suggest that HFO rate and amplitude are a function of electrode size, implying that these characteristics cannot be directly compared between HFOs recorded with electrodes of different sizes. This could represent a significant confounding factor in studies of HFOs as a predictive biomarker of surgical outcome. Further study is required to quantify and model this relationship and understand its clinical relevance for surgical planning.

## Supplementary Material

supp1-3416440

## Appendix

### Computational Model of HFO Measurement

#### A. HFO-Generation Process

Define a pulse train x(t) composed of L pulses, where the i-th pulse starts at $\tau_{0}^{\left(i\right)}$ and ends at $\tau_{1}^{\left(i\right)}$. An independent identically exponentially distributed random variable, $s_{i}$, is used to describe the gap time between the end of one pulse and the beginning of the next:

(1)
$$s_{i}=\tau_{0}^{\left(i\right)}-\tau_{1}^{\left(i-1\right)}\;\sim \;exp\left(\frac{1}{\frac{r_{\text{minute}}}{60}}\right)$$

where $r_{\text{minute}}$ is the average HFO rate per minute (Fig. 9(a)). The duration of each pulse, $d_{i}$, is then modeled with a random variable that follows a lognormal distribution:

(2)
$$d_{i}=\tau_{1}^{\left(i\right)}-\tau_{0}^{\left(i\right)}\;\sim \;lognorm\left(log\;\overset{‾}{d}-\frac{1}{2}\sigma_{d}^{2},\sigma_{d}^{2}\right)$$

where $\sigma_{d}^{2}$ is the variance of the duration and represents the skewness of the distribution, $\overset{‾}{d}$ is the average HFO duration in seconds (Fig. 9(b)), and $d_{i}$ is independent from any other $d_{j}$. Furthermore, each i-th pulse is assumed to have an amplitude $K_{i}$, which is modeled with a gamma distribution with shape $k_{K}=\theta_{K}^{-1}$ and scale $\theta_{K}=\sigma_{K}^{2}$ :

(3)
$$K_{i}\;\sim \;\Gamma \left(k_{K},\theta_{K}\right)$$

such that K has unit mean E[K]=1 (Fig. 9(c)). With an appropriate large shape (observed with a small standard deviation), it can be approximated by a normal distribution.

The distributions of neuronal parameters, such as firing rates and instantaneous amplitudes (that could be analogous to our pulse rates and envelopes), suggest skewed and heavy-tailed distributions [49], [50]. We are interested in modeling these factors using the proposed distributions. We have the conjecture that the amplitude distribution is skewed independently of the prior distribution of the amplitude K, especially for pair and quad electrodes. In our simulations, we define $\sigma_{d}=0.4$, $\overset{‾}{d}=0.040$, and $\sigma_{K}=0.2$.

Then, define a base HFO as a sinusoidal wave with frequency $f_{HFO}$ (in Hertz) and an envelope, $\vartheta_{HFO}(t)$ [51], [52], [53], described by a Gaussian function

(4)
$$\vartheta_{HFO}(t)=exp\left(-\frac{1}{2}{\left(\frac{t}{\sigma_{\vartheta }}\right)}^{2}\right)$$

with a scale $\sigma_{\vartheta }=\frac{1.5}{f_{\text{HFO}}}$ such that a sinusoid with this envelope will have at least four peaks with $\vartheta_{HFO}(t)>0.5$.

Then the HFO pulse train $x_{HFO}(t)$ is the convolution of the HFO envelope and the pulse train, multiplied with a sinusoidal with frequency $f_{HFO}$:

(5)
$$x_{HFO}(t)=\left(x(t)\;*\;\vartheta_{HFO}(t)\right)\;sin\;\left(2\pi f_{HFO}t\right)$$

This formulation also allows us to connect the pulse duration with the full-width at half-maximum [54].

#### B. Electrode-Tissue Interaction

We adopted the lumped model introduced in [30] that was used to simulate realistic HFO signals through combination with an electrode-tissue interaction (ETI) model and a 3D neural network model mimicking hippocampal recordings in rodent models. While its ETI model is also an adaptation of common circuit-equivalent representations [22], [55], [56], [57], [58], this model simulated fast ripples by introducing hyperexcitable neurons in the network and calculating a discretized net potential. Signals recorded from a mouse hippocampus under the Kainate model of temporal lobe epilepsy served to validate this computational simulation. In the current study, we adapt Al Harrach’s ETI and net potential model, which we will describe briefly.

ETI is modeled with an electrical equivalent circuit that contains an electrolyte resistance $R_{s}$ connected in series with a subcircuit. The subcircuit consists of a constant phase angle (CPA) impedance $Z_{CPA}$ and a charge transfer resistance (CTR) $R_{CT}$ arranged in parallel. The CTR component quantifies the charge leakage, and the CPA impedance represents non-Faradaic interactions across the electrode-tissue double layer. The CPA impedance is a non-linear component described as a function of the double-layer capacitance $C_{dl}$, approximated with $Z_{CPA}(\omega )={\left(C_{dl}j\omega^{n}\right)}^{-1}$ [30]. Consequently, the ETI equivalent impedance $Z_{eq}$ is

(6)
$$Z_{eq}=R_{s}+\frac{R_{CT}Z_{CPA}}{R_{CT}\;+\;Z_{CPA}}=R_{s}+\frac{R_{CT}}{R_{CT}C_{dl}j\omega^{n}\;+\;1}$$

Under the presence of a shunt capacitance $C_{s}$, the transfer function H(ω) is described by

(7)
$$H(\omega )={\left(1+j\omega Z_{eq}C_{s}\right)}^{-1}$$

The experimental values of the ETI circuit parameters for platinum electrodes approximately 1 mm in diameter are $R_{S}=1.22e3$, $R_{CT}=3.64e3$, $C_{dl}=3.17e-7$ and n=0.90 [59]. During our simulations, we define the shunt capacitance $C_{s}=100^{\sim }nF$ as in the suggested range for this component [30].

#### C. Two-Component Net Electrode Potential

The net potential V that the electrode measures is approximated as the average of the nearest P discretized extracellular point potentials $v_{p}$ over the electrode surface 𝒮 [30]:

(8)
$$V\left(t\right)={∯}_{a\in 𝒮}v_{p}\left(t\right)da=\frac{1}{P}\sum_{p=1}^{P}v_{p}\left(t\right)$$

Assume that the neurons in contact with the electrode belong to two categories: HFO-generating (HFOG) and the surrounding non HFO-generating (SURR) neurons. Therefore, the net potential can be expressed as

(9)
$$V(t)\;=\frac{1}{P}\left(\sum_{p=1}^{P_{SURR}}v_{p}^{\left(SURR\right)}(t)+\sum_{p=1}^{P_{HFOG}}v_{p}^{\left(HFOG\right)}(t)\right)\;\;=\frac{1}{P}\left(P_{SURR}V_{SURR}(t)+P_{HFOG}V_{HFOG}(t)\right)$$

where $P_{HFOG}$, $V_{HFOG}(t)$ and $P_{SURR}$, $V_{SURR}(t)$ are the number and the average potential of HFOG and SURR cells, respectively.

Because we are interested in the interplay between the surface area of the electrode and the area of the HFO-generating tissue, we can reformulate the total potential V(t) using the surface areas. Assuming that each neuron exposed to the electrode has the same area, we could also represent V(t) as

(10)
$$V(t)=\frac{1}{A_{E}}\left(A_{HFOG}V_{HFOG}(t)+A_{SURR}V_{SURR}(t)\right)$$

where $A_{E}$ is the total electrode surface area, with the total area of HFO-generating neurons $A_{HFOG}$ and the surrounding non HFO-generating neurons $A_{SURR}$.

In this study, we modeled $V_{SURR}(t)$ with a Gaussian white noise (GWN) process with variance $\sigma_{SURR}^{2}$. This stochastic model provides a reasonable representation of the behavior of a large, non-synchronized group of neurons. Assume that the signals generated by the SURR neurons $v_{p}^{\left(SURR\right)}$ are zero-mean, wide-sense stationary, and weakly dependent on time and space and in the Doukhan-Louhichi sense [60, Definition 1]. Then, the total potential converges to a normal distribution $V_{SURR}(\tau )|\;t=\tau \to 𝒩(0,\frac{1}{P_{SURR}}\sigma_{SURR}^{2})$ due to the Lindeberg Central Limit Theorem [61, Theorem 2], while it also is weakly dependent on time. Recall that a GWN process is zero-mean, normally distributed, and uncorrelated in time. Thus, due to the expected large number of SURR neurons, we can consider GWN as a suitable approximation model for $V_{SURR}(t)$.

Now, assume that each p-th HFOG neuron $v_{p}^{\left(HFOG\right)}(t)$ is the sum of two components: the HFO signal $x_{HFO}(t)$ described in Appendix Section A and a background signal $\varepsilon_{p}^{\left(HFOG\right)}(t)$. Thus, the total potential $V_{HFOG}$ will be the sum of the HFO signal and the average of the additional signals $E_{HFOG}(t)=\frac{1}{P_{HFOG}}\sum_{p=1}^{P_{HFOG}}\varepsilon_{p}^{\left(HFOG\right)}(t)$. Under a set of assumptions similar to those applied on the SURR neurons, we can also model the background signal $E_{p}(t)$ as a white noise process.

Based on the previous simplifications,

(11)
$$V(t)=\frac{1}{A_{E}}\left(A_{HFOG}x_{HFO}(t)+A_{HFOG}E_{HFOG}(t)\right.\;\;\left.+A_{SURR}V_{SURR}(t)\right)$$

where $E_{HFOG}(t)$ and $V_{SURR}(t)$ are modeled as zero-mean white noise processes with variances $\sigma_{HFOG}^{2}$ and $\sigma_{SURR}^{2}$. In our simulation, we use $\sigma_{HFOG}^{2}=\sigma_{SURR}^{2}=0.01$.

#### D. Spatial Spread of HFOs

Let $A_{0}$ be the area of a small electrode (1.07 mm^2^). Then, we can express $A_{E}$ as a multiple of the number of short-circuited electrodes $M:A_{E}=MA_{0}$, and we can also express the HFO-generating area as a proportion: $A_{HFOG}=\rho_{HFOG}A_{0}$.

The number of EEG channels simultaneously detecting a single HFO is often represented with a heavy-tailed distribution, including a geometric distribution [62]. Our results in Section III-E also support that hypothesis. But there is currently no mechanistic model to explain the spatial extent of HFOs, in part because any estimate of this characteristic is a function of the size of the electrodes used to do the measurement. Multiple sizes of electrodes could be used to address this, but there are inherent risks and limitations associated with the sequential implantation of multiple sets of electrodes in humans. In our theoretical model, we represent the spatial spread of HFOs by assuming that the proportion $\rho_{HFOG}$ is a gamma-distributed random variable:

(12)
$$\rho_{HFOG}\;\sim \;\Gamma \left(k_{HFOG},\frac{{\overset{‾}{\rho }}_{HFOG}}{k_{HFOG}}\right)$$

where $\Gamma \left(k_{HFOG},\theta_{HFOG}\right)$ is parameterized with $k_{HFOG}$ as the shape and a $\theta_{HFOG}=\frac{{\overset{‾}{\rho }}_{HFOG}}{k_{HFOG}}$ as the scale (Fig. 9(d)). Note that the average of $\rho_{HFOG}$ is ${\overset{‾}{\rho }}_{HFOG}$ and its standard variation is $\frac{{\overset{‾}{\rho }}_{HFOG}}{\sqrt{k_{HFOG}}}$. In our simulations, we define $k_{HFOG}=10$ and ${\overset{‾}{\rho }}_{HFOG}=0.75$.
