# Supplementary material for: Electrode Surface Area Impacts Measurement of High Frequency Oscillations in Human Intracranial EEG
Source: IEEE Trans Biomed Eng. Author manuscript; Available in PMC 2025 Jan 10. (PMC11723563; doi:10.1109/TBME.2024.3416440)
Supplement: supp1-3416440 [file NIHMS2031822-supplement-supp1-3416440.pdf]

Supplementary Information

Subj. No.	Age at Phase 2 (years)	Sex	Lesional Status and Etiology	Outcome (last follow-up)	Implanted Electrodes	Time post implant	SOZ in HD grid	Total duration of iEEG analyzed
1	15.4	F	Lesional; Left frontal focal cortical dysplasia	Seizure-free (48 months)	One HD 8x8, two 2x4, and one 4x8	Day 2, evening	39, 47, 55, 56, 62, 64	59 mins
2	11.9	M	Non-lesional; Unknown etiology; two seizure foci (right frontal and right temporal)	Seizure-free (36 months)	One HD 8x8, one 8x8, one 2x4, two 1x6, one 4x5, one 1x4; one 1x8 depth electrode	Day 2, evening	2, 10, 18, 25, 26, 33, 34	53 mins
3	19	M	Lesional; Left frontal traumatic brain injury	Seizure-free (16 months)	One HD 8x8, one 4x8, two 2x4, and one 2x8	Day 2, evening	27, 28, 36, 37, 43, 44	60 mins
4	16.7	F	Lesional; Perinatal stroke	Seizure-free (29 months)	One HD 8x8, one 8x8, two 4x8, one 2x6, one 1x6 and two 1x4	Day 3, evening	Not in HD grid	60 mins
5	6.8	F	Non-lesional; Unknown etiology	Seizure-free (26 months)	One HD 8x8, one 8x8, two 4x8 and two 2x6	Day 3, evening	No seizures captured	60 mins
6	1	M	Lesional; Large right frontal cortical dysplasia	Seizure-free (25 months)	One HD 8x8, one 8x8, two 1x6 and one 1x4	Day 3, evening	1- 64	60 mins
7	7.6	M	Lesional; Large right posterior cortical malformation	Seizure-free (24 months)	One HD 8x8, one 8x8, one 2x6, one 2x5, two 1x6 and one 1x4	Day 3, evening	27-30, 34-38, 43-47, 51-55, 58-62	60 mins
8	15.6	F	Lesional; Traumatic left fronto-temporo-parietal hemorrhage	Seizure-free (17 months)	One HD 8x8, one 8x8, one 2x4, two 1x6, and one 1x4	Day 2, evening	Not in HD grid	60 mins

Supplementary Table I: *Patient Information. Age is indicated in years, and all subjects had focal epilepsy. Implanted electrodes are subdural grids unless otherwise indicated. The column for time post-implant indicates when the iEEG recordings were done, with Day 1 defined as the day of implantation. The column for SOZ in HD grid indicates the number of each contact in the HD grid overlaying the SOZ. Abbreviations: male (M), female (F), high-density (HD).*

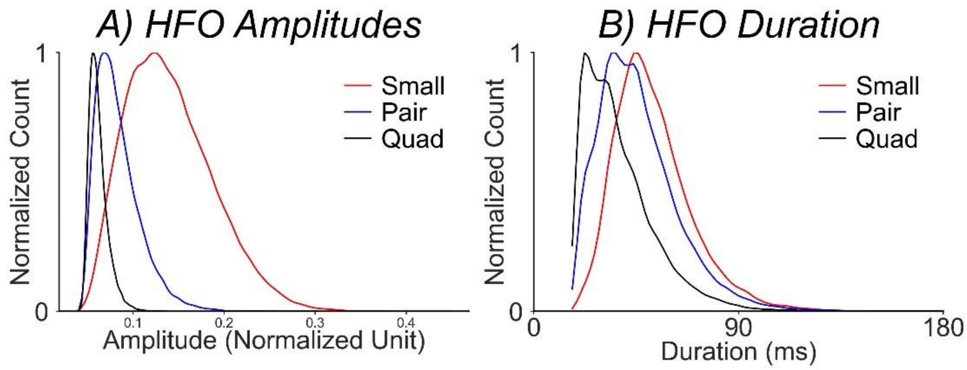

Fig. S1: *Simulation results for the HFO measurement model in the ripple frequency band (80-250 Hz). Histograms of* ***(A)*** *HFO amplitude and* ***(B)*** *HFO duration for small, pair, and quad electrodes.*

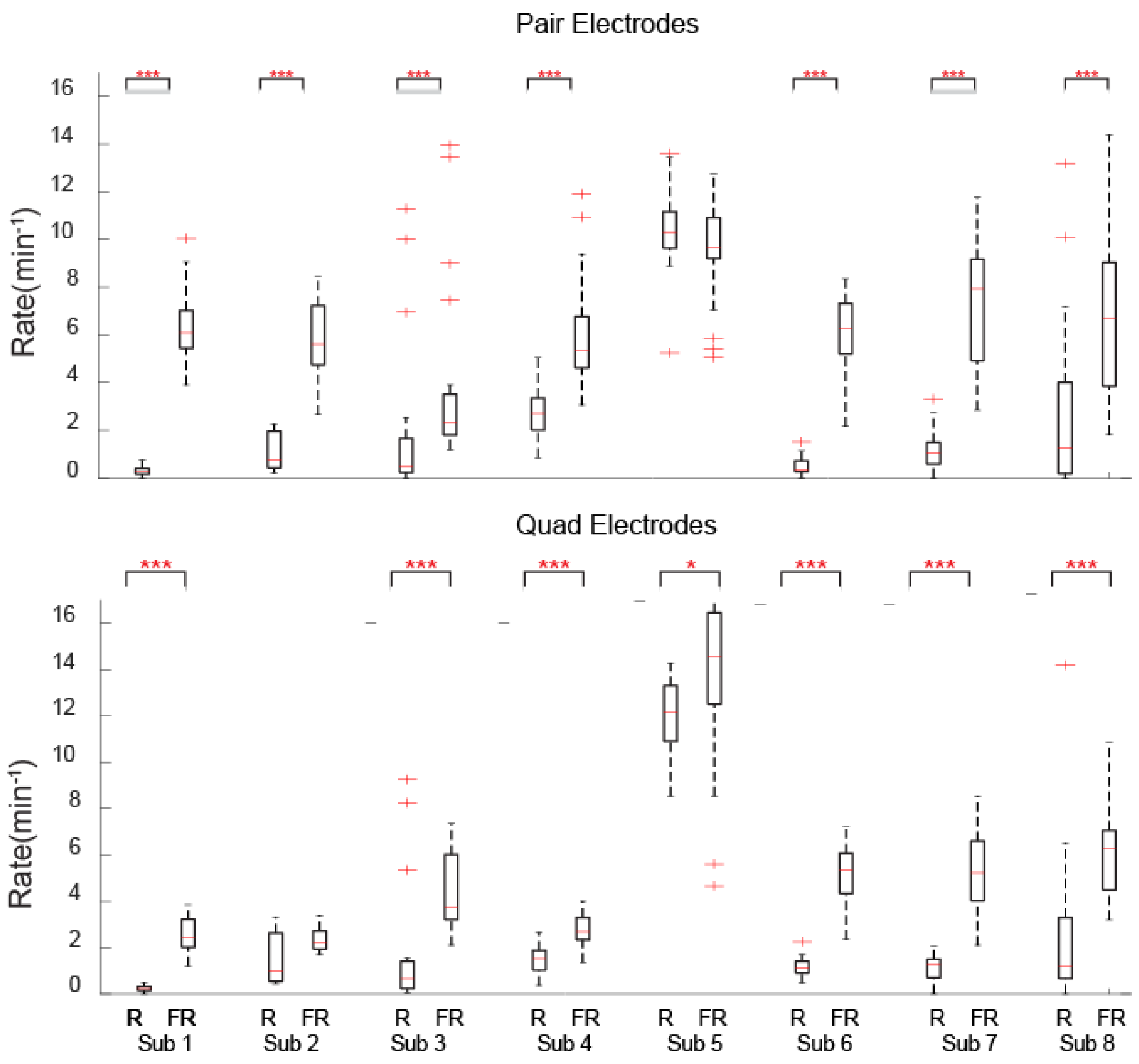

Fig. S2: *Boxplots showing the channel-wise HFO rates for pair (top) and quad (bottom) electrodes within each subject for ripples (R) and fast ripples (FR). * indicates p<0.05, ** indicates p<0.01 and *** indicates p<0.001.*

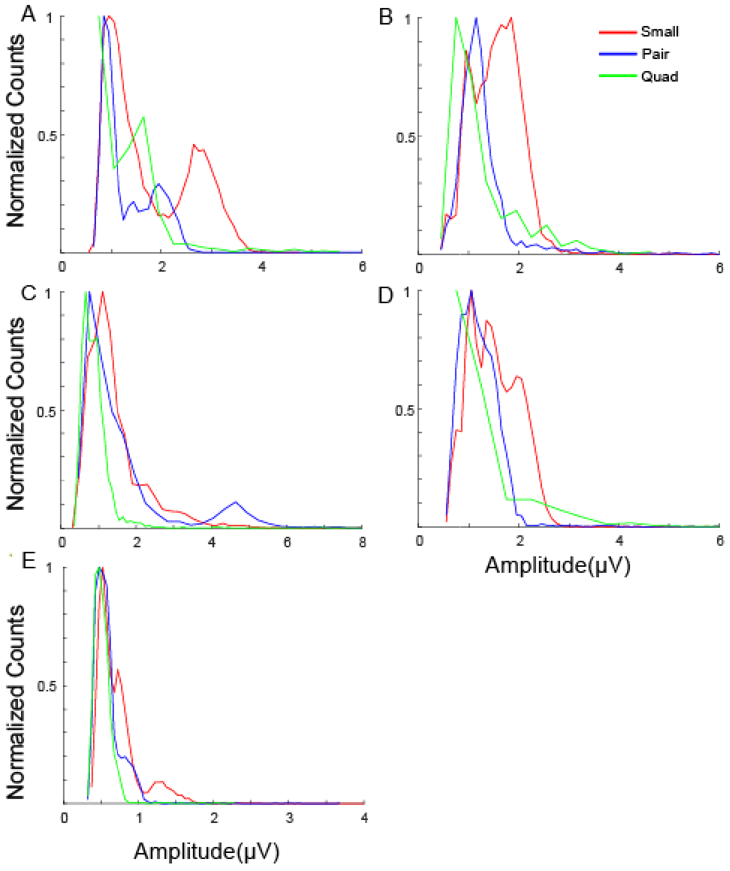

Fig. S3: *Histograms of fast ripple amplitude for the three electrode sizes. Results for the five subjects not shown in the main text are displayed here. The normalized counts in the histograms were obtained by dividing each value of count by the highest value within each property.*

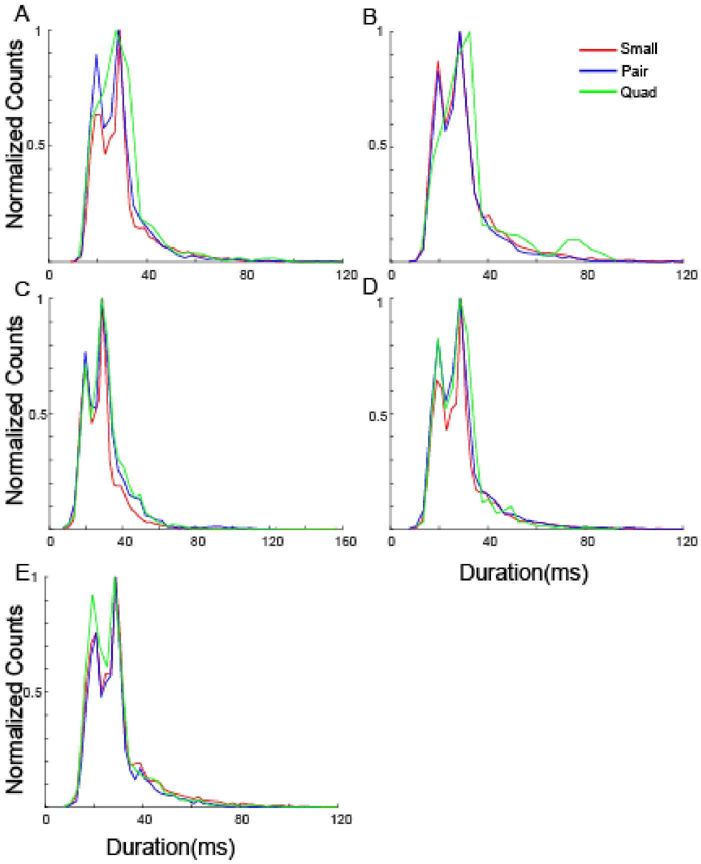

Fig. S4*: Histograms of fast ripple duration for the three electrode sizes. Results for the five subjects not shown in the main text are displayed here. The normalized counts in the histograms were obtained by dividing each value of count by the highest value within each property.*

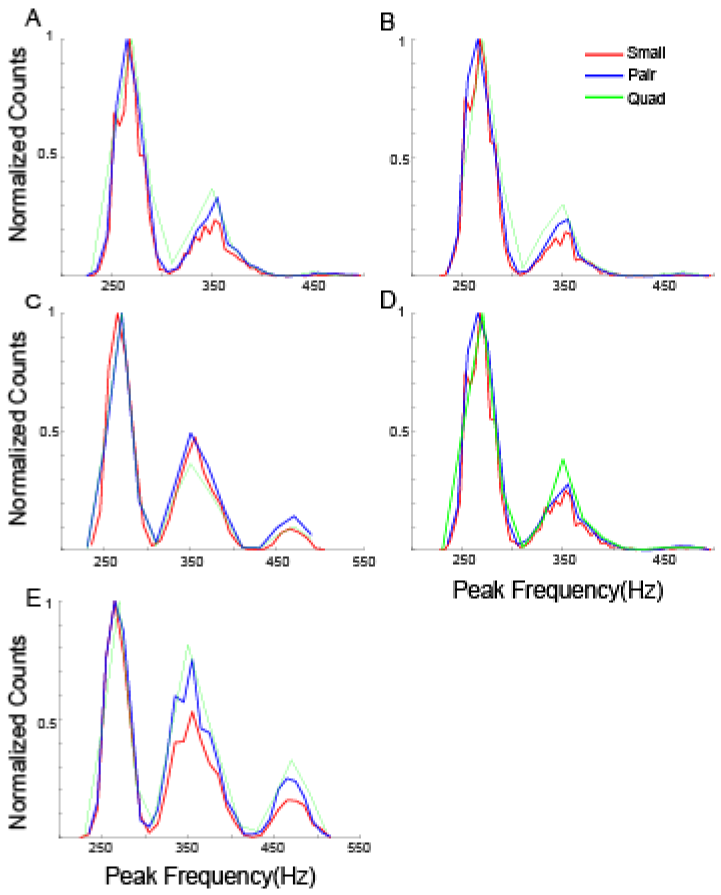

***Fig. S5**: Histograms of fast ripple peak frequency for the three electrode sizes. Results for the five subjects not shown in the main text are displayed here. The normalized counts in the histograms were obtained by dividing each value of count by the highest value within each property.*
